# Blood Microbiota and Its Products: Mechanisms of Interference with Host Cells and Clinical Outcomes

**DOI:** 10.3390/hematolrep16030043

**Published:** 2024-07-06

**Authors:** Luigi Santacroce, Ioannis Alexandros Charitos, Marica Colella, Raffaele Palmirotta, Emilio Jirillo

**Affiliations:** 1 Section of Microbiology and Virology, Interdisciplinary Department of Medicine, School of Medicine, University of Bari ‘Aldo Moro’, 70124 Bari, Italyraffaele.palmirotta@uniba.it (R.P.); emilio.jirillo@uniba.it (E.J.); 2 Istituti Clinici Scientifici Maugeri IRCCS, Pneumology and Respiratory Rehabilitation Unit, Institute of Bari, 70124 Bari, Italy; alexanestesia@hotmail.com; 3 Doctoral School, eCampus University, 22060 Novedrate, Italy

**Keywords:** bacteria, blood microbiota, dysbiosis: extracellular vesicles, immune cells, lipopolysaccharides, outer membrane vesicles

## Abstract

In healthy conditions, blood was considered a sterile environment until the development of new analytical approaches that allowed for the detection of circulating bacterial ribosomal DNA. Currently, debate exists on the origin of the blood microbiota. According to advanced research using dark field microscopy, fluorescent in situ hybridization, flow cytometry, and electron microscopy, so-called microbiota have been detected in the blood. Conversely, others have reported no evidence of a common blood microbiota. Then, it was hypothesized that blood microbiota may derive from distant sites, e.g., the gut or external contamination of blood samples. Alteration of the blood microbiota’s equilibrium may lead to dysbiosis and, in certain cases, disease. Cardiovascular, respiratory, hepatic, kidney, neoplastic, and immune diseases have been associated with the presence of Gram-positive and Gram-negative bacteria and/or their products in the blood. For instance, lipopolysaccharides (LPSs) and endotoxins may contribute to tissue damage, fueling chronic inflammation. Blood bacteria can interact with immune cells, especially with monocytes that engulf microorganisms and T lymphocytes via spontaneous binding to their membranes. Moreover, LPSs, extracellular vesicles, and outer membrane vesicles interact with red blood cells and immune cells, reaching distant organs. This review aims to describe the composition of blood microbiota in healthy individuals and those with disease conditions. Furthermore, special emphasis is placed on the interaction of blood microbiota with host cells to better understand disease mechanisms.

## 1. Introduction

For many years, in the absence of specific diseases, blood was considered a sterile environment. However, the development of new analytical approaches, i.e., real-time PCR, allowed the detection of bacterial ribosomal DNA in healthy individuals [1]. Moreover, using techniques such as dark field microscopy, fluorescent in situ hybridization, and flow cytometry, pleiomorphic bacteria have been identified in the blood, forming the so-called blood microbiota or microbiome [2]. The microbiota refers to bacteria, viruses, and fungi, while the microbiome is a collection of genomes or genomic fragments, such as the DNA and/or RNA of microorganisms. Moreover, light and electron microscopy have revealed that the blood microbiota undergoes a few transformations in the context of peripheral blood mononuclear cells (PBMCs), such as vesiculation, tubulation, budding, and the protrusion of progeny cells from large electron-dense bodies [3]. Current research on healthy blood microbiota has documented that the *Bacillota*, *Actinomycetota*, *Pseudomonadota*, and *Bacteroidota* phyla are dominant [4,5,6]. Microbial DNA detected in the blood of healthy subjects coexists in harmony with the host and exhibits immunomodulatory phenotypes. Their absence or presence may determine health or disease (sepsis) conditions [7]. Conversely, recent research based on the characterization of DNA signatures in the blood of 9.770 healthy individuals indicated no evidence of a common blood microbiota [8]. These findings suggest a transient process of commensal microbes’ translocation to the bloodstream from other sites. Others have suggested that blood microbiota may derive from microbial contamination in low-biomass samples and that the undetermined viability of blood microbiota depends on culture-independent procedures [9,10]. In this framework, there is a general debate on the source of blood microbiota. According to more recent views, it may directly derive from gut microbiota or the skin–oral–gut axis [11,12]. 

Maternal origin of the blood microbiota cannot be ruled out, since its presence has been found in certain prenatal tissues, such as umbilical cord blood, meconium, amnion, and the placenta [13]. In recent years, some research has suggested the hypothesis that a certain combination of bacteria in the placenta may have an important role in premature births. In addition, periodontal disease in pregnant women may be associated with an increased probability of premature births. This hypothesis, although intriguing in light of the well-known correlation between oral disease and preterm birth, also highlights the importance of oral hygiene during pregnancy as a preventative practice for pregnant women. According to more recent studies, the presence of a placental microbiota has been denied, whereas placental asepticity has been confirmed. These findings reinforce the idea that the fetus lives in a sterile environment that allows for metabolic exchanges with the maternal blood, recalling the hypothesis that first contact with bacteria occurs during childbirth through the birth canal.

Although this research requires more in-depth analysis and methodological refinement, the mechanisms behind the maternal transfer of blood microbiota are still unknown, despite proposals of oral and gut fetal compartment colonization or the fetal ingestion of amniotic fluid during gestation [14,15,16].

Interestingly, blood microbiota sequencing studies have demonstrated differences according to geographical areas. For instance, Germany and Poland are characterized by higher blood microbiota values, with intermediate levels seen in Italy and Finland, and lower levels in Belgium and Austria [17]. Differences in bacterial DNA distribution may rely on hosts’ genetic and immune factors or diet, hygienic factors, and parasitic loads [18,19,20]. It appears that the environment may have a greater impact than age, even if an association between blood microbiota and senescence cannot be excluded [21]. 

The persistence of microbes in the blood can cause several diseases involving the cardiovascular system, liver, and kidneys [21,22]. Compared to the gut, the blood microbiota can interact with the host to different extents, especially with leukocytes whose responses may determine disease status. Moreover, in healthy humans, the blood microbiota interacts with host cells via an array of products, e.g., metabolites, lipoglycans, quorum-sensing peptides, proteins, and bacterial extracellular vesicles (EVs) [23]. Outer membrane vesicles (OMVs) produced by Gram-negative bacteria are enriched in lipopolysaccharides (LPSs) and membrane proteins [24]. It is well known that LPSs are key molecules that interact with Toll-like receptors (TLR)-4 on monocytes and endothelial cells, activating the NF-kB pathway with the subsequent release of proinflammatory cytokines [25,26]. On the other hand, LPS micelles can be tolerated by the immune system, thus becoming innocuous to the host [27].

The disturbance of a core healthy microbiota in the blood may contribute to disease outcomes. In patients with myocardial infarction (MI) and chronic coronary syndrome, microbial diversity was reported to be higher than healthy individuals [28,29]. Liver fibrosis and cirrhosis are characterized by diverse blood microbiota. Certain bacteria provoke the release of proinflammatory cytokines and nitric oxide [30,31]. In kidney disease, circulating bacteria are not typical commensals of the urinary tract, thus suggesting their derivation from other sources, e.g., the gut [32,33]. In cancer, the blood microbiota profile helps distinguish different cancer types and predicts the response to advanced colon cancer [34,35]. In patients with autoimmune disease, immunosuppression, HIV, and inflammatory bowel disease (IBD), blood bacteria have also been detected. However, it is unclear whether bacteria are the causative agents of disease or are provoked by disease outcomes [36,37,38,39].

The objectives of the present review include describing the blood microbiota composition in either healthy individuals or sick subjects. Furthermore, illustrating blood microbiota/host cell interaction will help to better understand the pathogenic mechanisms elicited by blood microbiota.

## 2. Data Selection

A comprehensive analysis of the current literature, searching related biological and clinical data on Scopus, Clarivate Analytics/Web of Science, PubMed, and EMBASE, as well as using the ‘cited by’ and ‘similar articles’ options available in PubMed, was carried out to prepare this review. All relevant data, mainly from original articles and clinical trials, were extracted and reported after a critical appraisal process by two independent authors (I.A.C. and M.C.).

## 3. Healthy Blood Microbiota

The concept of blood microbiota has been accepted, though controversial. According to some researchers, the blood microbiota naturally exists from birth throughout life, consisting of unharmful organisms living in equilibrium with the host [40,41]. Others have identified circulating microbial cell-free DNA in the blood or microbial vesicles containing metabolites and fragmented DNA or RNA [42,43,44]. Conversely, other groups deny the existence of blood microbiota [45,46]. Researchers who support blood microbiota existence have no consensus on its composition. *Staphylococcus* spp. is a common genus detected in the blood, but information at the species level is very poor [47,48,49]. *Pseudomonadota* phylum and *Cutibacterium acnes* are also found in the blood [50,51]. Bacterial diversity varies between the buffy coat, red blood cells, and plasma with 117 blood microbial species, including 110 bacteria, 5 viruses, and 2 fungi. These data point to the absence of a core healthy blood microbiota, with transient and sporadic translocation of commensals into the circulation. These commensals are rapidly cleared out and do not colonize. Recently, electron microscopy has contributed to blood microbiota research. Circulating microbiota in PBMCs from healthy donors undergo complex life cycles, involving different morphological transformations [52]. Blood microbiota can reproduce by irregular binary fission, budding, protrusion–extrusion of progeny cells from large electron-dense bodies, vesiculation, tubulation, or a combination of all types. The morphology of blood microbiota supports the existence of microorganisms in the blood of healthy people.

Blood microbiota profiles are assessed in healthy individuals or in patients to identify genetic signatures for risk stratification, diagnosis, disease surveillance, as well as drug development. However, the existence of human blood microbiota is still debatable, considering two major issues: high-risk contamination in low-biomass samples and the undetermined viability of blood microbiota via culture-independent profiling methods [53].

## 4. Dysbiosis of Blood Microbiota and Disease Outcome

Many factors influence the composition of the blood microbiota, such as leaking epithelial junctions, mucosal disruption, periodontal disease, chewing, and tooth brushing [52,54,55]. Alteration of the blood microbiota equilibrium may result in dysbiosis, which, in turn, may cause several diseases and induce a state of low-grade inflammation. Chronic low-intensity inflammation is a condition defined in recent years as one that can develop pathophysiologically for a long period without symptoms and then trigger even serious diseases. Although there are no specific tests to allow diagnosis, suspicion is possible through careful collection of the patient’s medical history. In the following sections, blood bacteria in the pathogenesis of cardiovascular, respiratory, hepatic, and kidney diseases as well as cancers and immune diseases are discussed.

### 4.1. Cardiovascular Diseases

Bacteria, fragments, and their DNA have been identified in cardiovascular disease patients [56,57,58,59]. Importantly, LPS from blood bacteria participates in the atherosclerotic process via the formation of macrophage-derived foam cells [60,61,62].

*Pseudomonadota*, *Actinomycetota*, *Cyanobacteria*, and *Verrucomicrobia* from the circulating microbiota play a role in cardiovascular disease outcomes, with the Proteobacteria phylum predominant in acute coronary patients [63,64,65,66,67]. In light of the above findings, dysbiosis of the human blood microbiota has been proposed as a marker for cardiovascular disease prediction.

Blood *Desulfobacterota* is increased in acute coronary syndrome but decreased in chronic coronary syndrome. *Desulfobacterota* can reduce sulfur compounds, thus contributing to butyrate breakdown via the butyrate beta-oxidation pathway, maintaining the catabolic reaction’s equilibrium [68,69]. *Desulfobacterota* also releases LPS, promoting atherosclerotic progression.

Elevated amounts of both *Escherichia* and *Shigella*, associated with high levels of interleukin (IL)-8, have been detected in coronary artery disease patients [70]. LPS from these bacteria likely cause inflammation via TLR or the inflammasome pathway, contributing to the pathogenesis of chronic coronary syndrome [71].

A higher abundance of *Bifidobacterium* and reduced numbers of *Bacteroidota* were detected in MI patients’ blood [72]. MI patients’ diverse blood microbiota composition can be attributed to various factors, such as geographic region, type of diet, genetics, and environmental changes [73]. Of note, blood bacteria associated with cholesterol and lipid metabolism are reduced in MI patients, potentially favoring atherosclerosis [74]. Of note, among cholesterol-degrading bacteria belonging to the *Nocardiaceae* and *Aerocollaceae* families, *Gordonia*, *Propionibacterium*, *Chryseobacterium*, and *Rhodococcus* are the most common.

### 4.2. Respiratory Diseases

Studies on blood microbiota in respiratory disease are scarce. *Bacteroides*, *Alistipes*, *Parabacteroides*, and *Prevotella* are predominant in the blood, with a decrease in *Actinobacter*, *Verrucomicrobia,* and *Cyanobacteria* [75]. When patients are subgrouped according to inflammatory subtypes, lung function, and corticosteroid therapy, differential blood bacteria profiles allow accurate asthma diagnosis with elevated sensitivity and specificity. Moreover, through RNA-sequencing of peripheral blood samples, the genera *Acinetobacter*, *Serratia*, *Streptococcus*, and *Bacillus* have been associated with severe dyspnea in former and current smokers [76,77,78]. Interestingly, certain genera, such as *Streptococcus*, *Cutibacterium*, *Corynebacterium*, *Lactobacillus*, *Staphylococcus*, and *Bacillus* activate oxidative phosphorylation, mTOR, and Wnt/Beta-catenin pathways in circulating cells [79]. Similarly, blood *Escherichia coli*, *Bacillus* spp., *Campylobacter hominis*, *Pseudomonas* spp., *Thermoanaerobacter pseudethanolicus*, *Thermoanaerobacterium thermosaccharolyticum*, and *Staphylococcus epidermis* detection is correlated with severe COVID-19 [80,81,82].

### 4.3. Liver Diseases

Liver fibrosis and cirrhosis are responsible for the abundance of diverse gut-derived circulating bacteria [83,84]. In this respect, the genus *Bacteroides* and the family *Enterobateriaceae* are more elevated in the blood of cirrhotic patients than in healthy individuals [85,86].

From a pathogenic point of view, certain circulating bacteria, i.e., *Corynebacteriales*, are inversely associated with gamma-interferon, IL-17A, and tumor necrosis factor (TNF)-alpha and may predict the reversal of portal hypertension in hepatitis C virus (HCV)-induced cirrhosis upon termination of antiviral treatment [87]. The presence of circulating LPSs in HCV patients supports the above data, with resulting endotoxemia contributing to inflammatory damage via the release of proinflammatory cytokines [88,89,90,91,92].

### 4.4. Kidney Diseases

Evidence suggests that blood microbiota dysbiosis plays a role in chronic kidney disease (CKD). An inverse correlation between glomerular filtration rate and an increase in circulating *Pseudomonadota* has also been documented [93]. The genus *Devosia* in the blood appears to predict increased mortality risk in CKD patients on peritoneal dialysis with or without vascular calcification [94]. Furthermore, blood *Legionella* was elevated in IgA nephropathy patients, with putative involvement in kidney impairment and mortality [95]. It is worth noting that blood bacteria implicated in kidney diseases are not of urinary origin, suggesting that urinary mucosa alterations may not participate in blood microbiota dysbiosis [96].

### 4.5. Neoplastic Diseases

In a recent study, microbiome analysis of blood and tissue revealed sequence reads not mapped to the human genome; rather, they belong to microorganisms such as bacteria, archaea, and viruses [97]. In this context, the blood microbiota profile of cancer patients helped distinguish different cancer types at very early stages, e.g., hepatocellular carcinoma, myeloid cancer, and gastric cancer [98,99,100]. Furthermore, bacterial genetic material in the blood could predict the cancer therapy response. Among patients with advanced colorectal cancer being treated with oxaliplatin/capecitabine/adoptive T cell immunotherapy, responders exhibited lactobacilli and the genera *Bifidobacterium* and *Enterococcus* more abundantly than non-responders [101]. Similarly, the genera *Lewinella* and *Paludibaculum* predicted a better clinical response to nivolumab in a non-small cell lung carcinoma patient [102]. Specific beta-glucoronidase and/or beta-galactosidase microbes in the gut regulate estrogen metabolism and the so-called estrobolome. Their increase has been associated with a higher risk of estrogen receptor-positive breast cancer in post-menopausal women [103,104]. In the blood, beta-glucuronidase-producing bacteria were predominant in breast cancer patients, while beta-galactosidase bacteria were predominant in healthy subjects [105]. Importantly, treatment of estrogen-receptor positive breast tumor cells with tamoxifen, an anti-estrogen, and *Staphylococcus aureus* EVs suppressed the AKT and ERK oncogenic pathways, causing more cell death [106]. Evidence suggests that *Staphylococcus* is reduced in breast cancer patients. In summary, the reported data suggest that the blood microbiota may play a role in cancer diagnosis, prognosis, and therapy. 

### 4.6. Immune and Inflammatory Diseases

In autoimmune diseases, blood microbiota dysbiosis may play a pathogenic role. Elevated blood levels of the genera *Desulfoconvexum*, *Desulfofrigus*, *Desulfovibrio*, *Draconibacterium*, *Planococcus*, and *Psychrilyobacter* and the phylum *Gemmatimonadetes* have been identified in patients with systemic lupus erythematosus (SLE). They are also associated with plasma autoantibody levels [107]. In vitro experiments that exposed PBMCs from SLE patients to heat-inactivated *Planococcus* released IL-1 beta, IL-6, and TNF-alpha, suggesting that this bacterium maintains an inflammatory status in circulation [108]. Similarly, rheumatoid arthritis (RA) abundance in the blood of *Halomonas* and *Shewanella* may be implicated in joint inflammation [109]. Evidence suggests that RA is associated with increased gut permeability; therefore, microbial translocation to the blood may account for inflammation [110]. These data suggest that RA outcomes provoke blood microbiota dysbiosis; however, dysbiosis, once established, may fuel the inflammatory process and promote disease progression.

In immunosuppressed patients who received liver transplantation, the families *Microbacteriaceae*, *Nocardiaceae*, and *Anelloviridae* were increased with a decrease in *Enterobacteriaceae* [111,112]. Furthermore, an association between an increase in *Xanthomonadaceae* and *Enterobacteriaceae* growth and acute host-versus-graft rejection was reported [113]. Opportunistic bacteria growth may be a side effect of immunosuppressant therapy; therefore, research in this direction may contribute to organ transplantation success. In the blood of patients with HIV, an increase in many genera, such as *Veillonella*, *Massilia*, *Haemophilus*, *Arthrobacter*, and *Fusobacterium*, was observed. Furthermore, in vitro coculture of HIV+ PBMcs with *Massilia* and *Haemophilus* led to a massive release of proinflammatory cytokines [114]. These data suggest that in HIV patients’ blood, dysbiosis may exacerbate disease progression. In inflammatory bowel disease, plasma EVs aggravate the inflammatory status and increase *Escherichia/Shigella* numbers in the blood [115]. These data are supported by the notion that circulating LPSs have been detected in patients with IBD, aggravating pre-existing inflammatory conditions [116]. In a feces-induced peritonitis porcine model, the emergence of new circulating bacteria was revealed, including *Escherichia/Shigella*, *Staphylococcus*, *Cloacibacterium*, *Diaphorobacter*, *and Rhodanobacter* [117]. These bacteria are related to ABC transporters and oxidative phosphorylation, which may sustain the pathogenesis of peritonitis. Additionally, patients with acute pancreatitis have shown a severe depletion of the phylum *Actinobacteria* and an increase in *Bacteroidota* compared to healthy subjects [118]. In patients with large vessel vasculitis, changes in blood microbiota composition have been detected in terms of elevation of the classes *Cytophagia* and *Clostridia* compared to healthy subjects [119]. These data indicate the pathogenic role of blood microbes in host inflammatory conditions.

In Table 1, the predominant blood microbiota in various diseases is illustrated.

## 5. Mechanisms of Interaction in Blood Microbiota with Host Cells

The microbiota belonging to mucosal sites, e.g., the respiratory system, gut, and urogenital tract, interacts with several epithelial cell types, including immune cells. Conversely, blood microbes make contact with red blood cells and leukocytes only. Bacteria are engulfed by macrophages and polymorphonuclear cells before their destruction by lytic enzymes. On the other hand, spontaneous binding of Gram-negative and Gram-positive bacteria to T lymphocytes has been demonstrated in vitro and in vivo in patients with typhoid fever [117,118,119]. According to the intensity of the stimulus, both phagocytes and lymphocytes secrete various mediators that protect or damage the host. A weak stimulus cannot trigger immune reactions, so bacteria survive in full harmony with bacteria. Besides direct contact, bacteria mostly interact with PBMCs via their products. Once released into the bloodstream, LPS from the outer membrane of Gram-negative bacteria, lipoteichoic acid, peptidoglycans, and mycolic acids from Gram-positive bacteria activate immune cells via the production of proinflammatory cytokines and free radicals [120]. This process is upregulated in septicemia and endotoxemia characterized by elevated blood bacteria, as previously mentioned [121]. In this framework, microbiota-derived EVs have been detected in human blood from healthy volunteers [122]. They promote intercellular communication by carrying proteins, lipids, sugars, nucleic acids, and metabolites [123,124]. OMVs are an EV subtype produced by Gram-negative bacteria. They are natural proteo-liposomes with a double leaflet membrane containing bacterial metabolites, cytosolic proteins, and nucleic acids [125]. OMVs from *Pseudomonas aeruginosa* induce a potent innate immune response via LPS and protein components [126]. In healthy individuals, it has been hypothesized that OMVs derive from the gut following the disruption of epithelial cell tight junctions and then diffuse into the bloodstream, ultimately reaching distant organs [127]. LPS-bearing EVs have been detected via electron microscopy in leaky gut, and OMVs from *Bacteroides thetaiotamicron* can translocate from the intestinal epithelium to distant organs [128,129]. A recent study found that OMVs isolated from the red blood cells of healthy donors contained bacterial proteins and lipids [130]. The same study tracked the fusion of fluorescent *E. coli* EVs with PBMCs and discovered that EVs interacted with monocytes only. Furthermore, there is evidence that LPSs from *E. coli* OMVs can interact with TLR4 in monocytes but not with T cells, B cells, or γδ-2 unconventional T cells. These data suggest that bacterial EVs participate in the interaction between the blood microbiota and host cells, leading to the activation of the NF-kB pathway and the subsequent release of proinflammatory cytokines and free radicals. 

**Figure 1:**
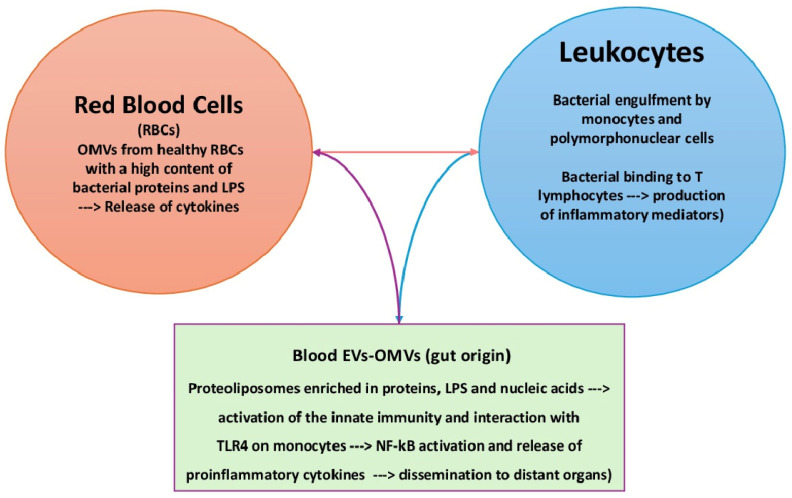
Interaction of the blood microbiota with host cells. Blood bacteria can directly interact with monocytes (engulfment) or T lymphocytes (binding). Bacterial products, e.g., EVs and OMVs, contain toxic products, such as LPSs, which can stimulate innate immunity and interact with TLR4 on monocytes. EVs: extracellular vesicles; OMVs: outer membrane vesicles.

The main mechanisms of the interaction between the blood microbiota and host cells are depicted in Figure 1.

Based on the data reported above, we can clearly affirm that systemic dysbiosis accounts for many pathologic conditions mediated by blood microbes. Conversely, commensal microbiota and restored eubiosis play an important role in host recovery.

## 6. Conclusions

Research on the blood microbiota requires further investigation since data are scarce and, sometimes, controversial. For instance, studies based on the bacteriome do not consider the viriome, archaeome, or mycobiome. Research on the blood microbiota is limited by these examples. To better understand the pathogenesis of various human diseases, the relationship between microorganisms from different kingdoms must be investigated. Current investigations into blood core bacteria are based on observational studies only, which indicate correlations but not causal relationships. Consequently, additional exploration should be carried out to decipher the disease mechanisms elicited by these core bacteria. Moreover, they originate from the host commensals, and diverse core bacteria have been observed in the same disease. In this regard, contrasting results about the existence and origin of fetal/placental microbiota, as determined by different sampling techniques and study methods, offer interesting definitions for the unborn child’s microbiota acquisition mechanisms, the infection mechanisms in utero, and their consequences.

These findings suggest that diverse core microbiota may contribute to the pathogenesis of common diseases, indicating a redundancy of microbes in different communities, with a convergent evolution when exposed to similar environments. Therefore, pooling and meta-analyzing data from independent studies with appropriate adjustments to confounding variables is needed. In conclusion, evidence supporting a core healthy blood microbiota is still limited. However, microbial gene signatures may be of diagnostic, prognostic, and therapeutic value, prompting further studies and better validation of current data.

## Figures and Tables

**Table 1:** The components of blood microbiota associated with various diseases in humans.

Blood Microbiota and Pathologic Conditions	
DISEASES	Associate Bacterial Species
CARDIOVASCULAR	- *Pseudomonadota* and *Desulfobacteria* ----> coronary disease; - *Escherichia-Shigella* ----> coronary disease ---> IL-8; - Increase in *Bifidobacterium* and decrease in *Bacteroidota* and bacteria associated with cholesterol and lipid excess in myocardial infarction;
RESPIRATORY	- Increase in *Bacteroides*, *Alistipes*, *Parabacteroides*, and *Prevotella*, and a decrease in *Actinobacter*, *Verrucomicrobia,* and *Cyanobacteria;*
LIVER	- Increase in *Bacteroides* and *Enterobacteriaceae* in cirrhotic patients; - Increase in *Corynebacteriales* ----> reversal of portal hypertension in HCV patients;
KIDNEY	- *Devosia* products ----> increase in mortality risk in CKD patients; - Increase in *Legionella* spp. in IgA nephropathy patients;
NEOPLASTIC	- Increase in *Bifidobacterium* and *Enterococcus* ----> distinction of different cancer types and prediction of response to therapy; - Increase of beta-glucuronidase-producing bacteria in breast cancer patients;
IMMUNE	- Increase in *Desulfoconvexum*, *Desulfofrigus*, *Draconibacterium*, *Planococcus,* and *Psychrilyobacter* in SLE patients; - Increase in *Halomonas* and *Shewanella* in RA patients; - Increase in *Anelloviridae*, *Nocardiaceae*, and *Microbacteriaceae* and decrease in *Enterobacteriaceae* in liver transplanted patients; - Increase in *Veillonella*, *Massilia*, *Haemophilus*, *Arthrobacter,* and *Fusobacterium* ----> proinflammatory cytokine release in HIV patients; - Increase in *Escherichia/Shigella* in IBD.

Abbreviations: HCV: Hepatitis C Virus; CKD: Chronic Kidney Disease; SLE Systemic Lupus Erythematosus; RA: Rheumatoid Arthritis; HIV: Human Immunodeficiency Virus; IBD: Inflammatory Bowel Disease.

## Data Availability

All available data have been reported in the manuscript.

## Abbreviations

CKD	Chronic Kidney Disease
EVs	Extracellular Vesicles
HCV	Hepatitis C Virus
IL	Interleukin
LPS	Lipopolysaccharides
MI	Myocardial Infarction
NO	Nitric Oxide
OMVs	Outer Membrane Vesicles
PBMCs	Peripheral Blood Mononuclear Cells
RA	Rheumatoid Arthritis
SLE	Systemic Lupus Erythematosus
TLR	Toll-like receptor
TNF	Tumor Necrosis Factor

## References

[1] Nikkari S., McLaughlin I.J., Bi W., Dodge D.E., Relman D.A. (2001). Does blood of healthy subjects contain bacterial ribosomal DNA?. J. Clin. Microbiol..

[2] McLaughlin R.W., Vali H., Lau P.C., Palfree R.G., De Ciccio A., Sirois M., Ahmad D., Villemur R., Desrosiers M., Chan E.C. (2002). Are there naturally occurring pleomorphic bacteria in the blood of healthy humans?. J. Clin. Microbiol..

[3] Tsafarova B., Hodzhev Y., Yordanov G., Tolchkov V., Kalfin R., Panaiotov S. (2023). Morphology of blood microbiota in healthy individuals assessed by light and electron microscopy. Front. Cell. Infect. Microbiol..

[4] Khan I., Khan I., Jianye Z., Xiaohua Z., Khan M., Hilal M.G., Kakakhel M.A., Mehmood A., Lizhe A., Zhiqiang L. (2022). Exploring blood microbial communities and their influence on human cardiovascular disease. J. Clin. Lab. Anal..

[5] Eisenhofer R., Minich J.J., Marotz C., Cooper A., Knight R., Weyrich L.S. (2019). Contamination in Low Microbial Biomass Microbiome Studies: Issues and Recommendations. Trends Microbiol..

[6] Santacroce L., Bottalico L., Charitos I.A. (2020). The Impact of COVID-19 on Italy: A Lesson for the Future. Int. J. Occup. Environ. Med..

[7] Tan C.C.S., Trew J., Peacock T.P., Mok K.Y., Hart C., Lau K., Ni D., Orme C.D.L., Ransome E., Pearse W.D. (2023). Genomic screening of 16 UK native bat species through conservationist networks uncovers coronaviruses with zoonotic potential. Nat. Commun..

[8] Santacroce L., Charitos I.A., Carretta D.M., De Nitto E., Lovero R. (2021). The human coronaviruses (HCoVs) and the molecular mechanisms of SARS-CoV-2 infection. J. Mol. Med..

[9] Potgieter M., Bester J., Kell D.B., Pretorius E. (2015). The dormant blood microbiome in chronic, inflammatory diseases. FEMS Microbiol. Rev..

[10] Velmurugan G., Dinakaran V., Rajendhran J., Swaminathan K. (2020). Blood Microbiota and Circulating Microbial Metabolites in Diabetes and Cardiovascular Disease. Trends Endocrinol. Metab..

[11] Castillo D.J., Rifkin R.F., Cowan D.A., Potgieter M. (2019). The Healthy Human Blood Microbiome: Fact or Fiction?. Front. Cell. Infect. Microbiol..

[12] Santacroce L., Man A., Charitos I.A., Haxhirexha K., Topi S. (2021). Current knowledge about the connection between health status and gut microbiota from birth to elderly. A narrative review. Front. Biosci..

[13] Romano-Keeler J., Sun J. (2022). The First 1000 Days: Assembly of the Neonatal Microbiome and Its Impact on Health Outcomes. Newborn.

[14] Perez-Muñoz M.E., Arrieta M.C., Ramer-Tait A.E., Walter J. (2017). A critical assessment of the “sterile womb” and “in utero colonization” hypotheses: Implications for research on the pioneer infant microbiome. Microbiome.

[15] D’Aquila P., Giacconi R., Malavolta M., Piacenza F., Bürkle A., Villanueva M.M., Dollé M.E.T., Jansen E., Grune T., Gonos E.S. (2021). Microbiome in Blood Samples From the General Population Recruited in the MARK-AGE Project: A Pilot Study. Front. Microbiol..

[16] Gupta V.K., Paul S., Dutta C. (2017). Geography, Ethnicity or Subsistence-Specific Variations in Human Microbiome Composition and Diversity. Front. Microbiol..

[17] Topi S., Bottalico L., Charitos I.A., Colella M., Di Domenico M., Palmirotta R., Santacroce L. (2022). Biomolecular Mechanisms of Autoimmune Diseases and Their Relationship with the Resident Microbiota: Friend or Foe?. Pathophysiology.

[18] Mobeen F., Sharma V., Tulika P. (2018). Enterotype Variations of the Healthy Human Gut Microbiome in Different Geographical Regions. Bioinformation.

[19] Amar J., Lange C., Payros G., Garret C., Chabo C., Lantieri O., Courtney M., Marre M., Charles M.A., Balkau B. (2013). Blood microbiota dysbiosis is associated with the onset of cardiovascular events in a large general population: The D.E.S.I.R. study. PLoS ONE.

[20] Shah N.B., Allegretti A.S., Nigwekar S.U., Kalim S., Zhao S., Lelouvier B., Servant F., Serena G., Thadhani R.I., Raj D.S. (2019). Blood Microbiome Profile in CKD: A Pilot Study. Clin. J. Am. Soc. Nephrol..

[21] Chronopoulos A., Kalluri R. (2020). Emerging role of bacterial extracellular vesicles in cancer. Oncogene.

[22] Schaack B., Hindré T., Quansah N., Hannani D., Mercier C., Laurin D. (2022). Microbiota-Derived Extracellular Vesicles Detected in Human Blood from Healthy Donors. Int. J. Mol. Sci..

[23] Magrone T., Jirillo E. (2011). The impact of bacterial lipolysaccharides on the endothelial system: Pathological consequences and therapeutic countermeasures. Endocr. Metab. Immune Disord.-Drug Targets.

[24] Konig M.F. (2020). The microbiome in autoimmune rheumatic disease. Best Pract. Res. Clin. Rheumatol..

[25] Magrone T., Jirillo E. (2019). The Tolerant Immune System: Biological Significance and Clinical Implications of T Cell Tolerance. Endocr. Metab. Immune Disord.-Drug Targets.

[26] Amar J., Lelouvier B., Servant F., Lluch J., Burcelin R., Bongard V., Elbaz M. (2019). Blood Microbiota Modification after Myocardial Infarction Depends upon Low-Density Lipoprotein Cholesterol Levels. J. Am. Heart Assoc..

[27] Khan I., Khan I., Usman M., Jianye Z., Wei Z.X., Ping X., Zhiqiang L., Lizhe A. (2022). Analysis of the blood bacterial composition of patients with acute coronary syndrome and chronic coronary syndrome. Front. Cell. Infect. Microbiol..

[28] Gedgaudas R., Bajaj J.S., Skieceviciene J., Varkalaite G., Jurkeviciute G., Gelman S., Valantiene I., Zykus R., Pranculis A., Bang C. (2022). Circulating microbiome in patients with portal hypertension. Gut Microbes.

[29] Kim Y., Carrai M., Leung M.H.Y., Chin J., Li J., Lee P.K.H., Beatty J.A., Pfeiffer D.U., Barrs V.R. (2021). Dysbiosis of the Urinary Bladder Microbiome in Cats with Chronic Kidney Disease. mSystems.

[30] Perez-Carrasco V., Soriano-Lerma A., Soriano M., Gutiérrez-Fernández J., Garcia-Salcedo J.A. (2021). Urinary Microbiome: Yin and Yang of the Urinary Tract. Front. Cell. Infect. Microbiol..

[31] Worby C.J., Schreiber HL 4th Straub T.J., van Dijk L.R., Bronson R.A., Olson B.S., Pinkner J.S., Obernuefemann C.L.P., Muñoz V.L., Paharik A.E., Azimzadeh P.N. (2022). Longitudinal multi-omics analyses link gut microbiome dysbiosis with recurrent urinary tract infections in women. Nat. Microbiol..

[32] Poore G.D., Kopylova E., Zhu Q., Carpenter C., Fraraccio S., Wandro S., Kosciolek T., Janssen S., Metcalf J., Song S.J. (2020). Microbiome analyses of blood and tissues suggest cancer diagnostic approach. Nature.

[33] Guo H., Li B., Diao L., Wang H., Chen P., Jiang M., Zhao L., He Y., Zhou C. (2021). An immune-based risk-stratification system for predicting prognosis in pulmonary sarcomatoid carcinoma (PSC). Oncoimmunology.

[34] Luo Z., Alekseyenko A.V., Ogunrinde E., Li M., Li Q.Z., Huang L., Tsao B.P., Kamen D.L., Oates J.C., Li Z. (2021). Rigorous Plasma Microbiome Analysis Method Enables Disease Association Discovery in Clinic. Front. Microbiol..

[35] Audo R., Sanchez P., Rivière B., Mielle J., Tan J., Lukas C., Macia L., Morel J., Immediato Daien C. (2022). Rheumatoid arthritis is associated with increased gut permeability and bacterial translocation which are reversed by inflammation control. Rheumatology.

[36] Kato K., Nagao M., Miyamoto K., Oka K., Takahashi M., Yamamoto M., Matsumura Y., Kaido T., Uemoto S., Ichiyama S. (2017). Longitudinal Analysis of the Intestinal Microbiota in Liver Transplantation. Transplant. Direct.

[37] Sharma A., Giorgakis E. (2022). Gut microbiome dysbiosis in the setting of solid organ transplantation: What we have gleaned from human and animal studies. World J. Transplant..

[38] Markova N.D. (2017). L-form bacteria cohabitants in human blood: Significance for health and diseases. Discov. Med..

[39] Dimova T., Terzieva A., Djerov L., Dimitrova V., Nikolov A., Grozdanov P., Markova N. (2017). Mother-to-newborn transmission of mycobacterial L-forms and Vδ2 T-cell response in placentobiome of BCG-vaccinated pregnant women. Sci. Rep..

[40] Zozaya-Valdés E., Wong S.Q., Raleigh J., Hatzimihalis A., Ftouni S., Papenfuss A.T., Sandhu S., Dawson M.A., Dawson S.J. (2021). Detection of cell-free microbial DNA using a contaminant-controlled analysis framework. Genome Biol..

[41] Wang S.E. (2020). Extracellular Vesicles and Metastasis. Cold Spring Harb. Perspect. Med..

[42] Ricci V., Carcione D., Messina S., Colombo G.I., D’Alessandra Y. (2020). Circulating 16S RNA in Biofluids: Extracellular Vesicles as Mirrors of Human Microbiome?. Int. J. Mol. Sci..

[43] Martel J., Wu C.Y., Huang P.R., Cheng W.Y., Young J.D. (2017). Pleomorphic bacteria-like structures in human blood represent non-living membrane vesicles and protein particles. Sci. Rep..

[44] Tan C.C.S., Ko K.K.K., Chen H., Liu J., Loh M., Chia M., Nagarajan N., SG10K_Health Consortium (2023). No evidence for a common blood microbiome based on a population study of 9,770 healthy humans. Nat. Microbiol..

[45] Santacroce L., Cagiano R., Del Prete R., Bottalico L., Sabatini R., Carlaio R.G., Prejbeanu R., Vermesan H., Dragulescu S.I., Vermesan D. (2008). Helicobacter pylori infection and gastric MALTomas: An up-to-date and therapy highlight. Clin. Ther..

[46] Païssé S., Valle C., Servant F., Courtney M., Burcelin R., Amar J., Lelouvier B. (2016). Comprehensive description of blood microbiome from healthy donors assessed by 16S targeted metagenomic sequencing. Transfusion.

[47] Damgaard C., Magnussen K., Enevold C., Nilsson M., Tolker-Nielsen T., Holmstrup P., Nielsen C.H. (2015). Viable bacteria associated with red blood cells and plasma in freshly drawn blood donations. PLoS ONE.

[48] Lluch J., Servant F., Païssé S., Valle C., Valière S., Kuchly C., Vilchez G., Donnadieu C., Courtney M., Burcelin R. (2015). The Characterization of Novel Tissue Microbiota Using an Optimized 16S Metagenomic Sequencing Pipeline. PLoS ONE.

[49] Zhai T., Ren W., Ji X., Wang Y., Chen H., Jin Y., Liang Q., Zhang N., Huang J. (2024). Distinct compositions and functions of circulating microbial DNA in the peripheral blood compared to fecal microbial DNA in healthy individuals. mSystems.

[50] Tong X., Yu X., Du Y., Su F., Liu Y., Li H., Liu Y., Mu K., Liu Q., Li H. (2022). Peripheral Blood Microbiome Analysis via Noninvasive Prenatal Testing Reveals the Complexity of Circulating Microbial Cell-Free DNA. Microbiol. Spectr..

[51] Delzenne N.M., Neyrinck A.M., Bäckhed F., Cani P.D. (2011). Targeting gut microbiota in obesity: Effects of prebiotics and probiotics. Nat. Rev. Endocrinol..

[52] Cheng H.S., Tan S.P., Wong D.M.K., Koo W.L.Y., Wong S.H., Tan N.S. (2023). The Blood Microbiome and Health: Current Evidence, Controversies, and Challenges. Int. J. Mol. Sci..

[53] Dickson R.P., Huffnagle G.B. (2015). The Lung Microbiome: New Principles for Respiratory Bacteriology in Health and Disease. PLoS Pathog..

[54] Moreno C.M., Boeree E., Freitas C.M.T., Weber K.S. (2023). Immunomodulatory role of oral microbiota in inflammatory diseases and allergic conditions. Front. Allergy.

[55] Könönen E., Gursoy M., Gursoy U.K. (2019). Periodontitis: A Multifaceted Disease of Tooth-Supporting Tissues. J. Clin. Med..

[56] Mansour S., Alkhaaldi S.M.I., Sammanasunathan A.F., Ibrahim S., Farhat J., Al-Omari B. (2024). Precision Nutrition Unveiled: Gene-Nutrient Interactions, Microbiota Dynamics, and Lifestyle Factors in Obesity Management. Nutrients.

[57] Tang H., Huang Y., Yuan D., Liu J. (2024). Atherosclerosis, gut microbiome, and exercise in a meta-omics perspective: A literature review. PeerJ.

[58] Chen Z., Lin Y., Wang J., Yao K., Xie Y., Chen X., Zhou T. (2024). Relationship between Compound α-Ketoacid and Microinflammation in Patients with Chronic Kidney Disease. Curr. Pharm. Des..

[59] Gutierrez P.S. (2022). Foam Cells in Atherosclerosis. Arq. Bras. Cardiol..

[60] Tabas I., Lichtman A.H. (2017). Monocyte-Macrophages and T Cells in Atherosclerosis. Immunity.

[61] An D., Hao F., Hu C., Kong W., Xu X., Cui M.Z. (2018). JNK1 Mediates Lipopolysaccharide-Induced CD14 and SR-AI Expression and Macrophage Foam Cell Formation. Front. Physiol..

[62] Khan I., Khan I., Kakakhel M.A., Xiaowei Z., Ting M., Ali I., Fei Y., Jianye Z., Zhiqiang L., Lizhe A. (2022). Comparison of Microbial Populations in the Blood of Patients With Myocardial Infarction and Healthy Individuals. Front. Microbiol..

[63] Hillman E.T., Lu H., Yao T., Nakatsu C.H. (2017). Microbial Ecology along the Gastrointestinal Tract. Microbes Environ..

[64] Huse S.M., Dethlefsen L., Huber J.A., Mark Welch D., Relman D.A., Sogin M.L. (2008). Exploring microbial diversity and taxonomy using SSU rRNA hypervariable tag sequencing. PLoS Genet..

[65] Amar J., Serino M., Lange C., Chabo C., Iacovoni J., Mondot S., Lepage P., Klopp C., Mariette J., Bouchez O. (2011). Involvement of tissue bacteria in the onset of diabetes in humans: Evidence for a concept. Diabetologia.

[66] Rajendhran J., Shankar M., Dinakaran V., Rathinavel A., Gunasekaran P. (2013). Contrasting circulating microbiome in cardiovascular disease patients and healthy individuals. Int. J. Cardiol..

[67] Yu I., Wu R., Tokumaru Y., Terracina K.P., Takabe K. (2022). The Role of the Microbiome on the Pathogenesis and Treatment of Colorectal Cancer. Cancers.

[68] Hao L., Michaelsen T.Y., Singleton C.M., Dottorini G., Kirkegaard R.H., Albertsen M., Nielsen P.H., Dueholm M.S. (2020). Novel syntrophic bacteria in full-scale anaerobic digesters revealed by genome-centric metatranscriptomics. ISME J..

[69] Sears C.L., Salzberg S.L. (2020). Microbial Diagnostics for Cancer: A Step Forward but Not Prime Time Yet. Cancer Cell.

[70] Arab J.P., Martin-Mateos R.M., Shah V.H. (2018). Gut-liver axis, cirrhosis and portal hypertension: The chicken and the egg. Hepatol. Int..

[71] Zhou X., Li J., Guo J., Geng B., Ji W., Zhao Q., Li J., Liu X., Liu J., Guo Z. (2018). Gut-dependent microbial translocation induces inflammation and cardiovascular events after ST-elevation myocardial infarction. Microbiome.

[72] Colella M., Charitos I.A., Ballini A., Cafiero C., Topi S., Palmirotta R., Santacroce L. (2023). Microbiota revolution: How gut microbes regulate our lives. World J. Gastroenterol..

[73] Ullah Goraya M., Li R., Gu L., Deng H., Wang G. (2023). Blood Stream Microbiota Dysbiosis Establishing New Research Standards in Cardio-Metabolic Diseases, A Meta-Analysis Study. Microorganisms.

[74] Lee J.H., Choi J.P., Yang J., Won H.K., Park C.S., Song W.J., Kwon H.S., Kim T.B., Kim Y.K., Park H.S. (2020). Metagenome analysis using serum extracellular vesicles identified distinct microbiota in asthmatics. Sci. Rep..

[75] Kozak M., Pawlik A. (2023). The Role of the Oral Microbiome in the Development of Diseases. Int. J. Mol. Sci..

[76] Shapiro H., Goldenberg K., Ratiner K., Elinav E. (2022). Smoking-induced microbial dysbiosis in health and disease. Clin. Sci..

[77] Dein Terra Mota Ribeiro A.B., Heimesaat M.M., Bereswill S. (2017). Changes of the Intestinal Microbiome-Host Homeostasis in HIV-Infected Individuals—A Focus on the Bacterial Gut Microbiome. Eur. J. Microbiol. Immunol..

[78] Dereschuk K., Apostol L., Ranjan I., Chakladar J., Li W.T., Rajasekaran M., Chang E.Y., Ongkeko W.M. (2021). Identification of Lung and Blood Microbiota Implicated in COVID-19 Prognosis. Cells.

[79] Tanacli R., Doeblin P., Götze C., Zieschang V., Faragli A., Stehning C., Korosoglou G., Erley J., Weiss J., Berger A. (2021). COVID-19 vs. Classical Myocarditis Associated Myocardial Injury Evaluated by Cardiac Magnetic Resonance and Endomyocardial Biopsy. Front. Cardiovasc. Med..

[80] Lovero R., Charitos I.A., Topi S., Castellaneta F., Cazzolla A.P., Colella M. (2023). Current Views about the Link between SARS-CoV-2 and the Liver: Friends or Foe?. Endocr. Metab. Immune Disord.-Drug Targets.

[81] Kajihara M., Koido S., Kanai T., Ito Z., Matsumoto Y., Takakura K., Saruta M., Kato K., Odamaki T., Xiao J.Z. (2019). Characterisation of blood microbiota in patients with liver cirrhosis. Eur. J. Gastroenterol. Hepatol..

[82] Lelouvier B., Servant F., Païssé S., Brunet A.C., Benyahya S., Serino M., Valle C., Ortiz M.R., Puig J., Courtney M. (2016). Changes in blood microbiota profiles associated with liver fibrosis in obese patients: A pilot analysis. Hepatology.

[83] Santiago A., Pozuelo M., Poca M., Gely C., Nieto J.C., Torras X., Román E., Campos D., Sarrabayrouse G., Vidal S. (2016). Alteration of the serum microbiome composition in cirrhotic patients with ascites. Sci. Rep..

[84] Virseda-Berdices A., Brochado-Kith O., Díez C., Hontañon V., Berenguer J., González-García J., Rojo D., Fernández-Rodríguez A., Ibañez-Samaniego L., Llop-Herrera E. (2022). Blood microbiome is associated with changes in portal hypertension after successful direct-acting antiviral therapy in patients with HCV-related cirrhosis. J. Antimicrob. Chemother..

[85] Jirillo E., Caccavo D., Magrone T., Piccigallo E., Amati L., Lembo A., Kalis C., Gumenscheimer M. (2002). The role of the liver in the response to LPS: Experimental and clinical findings. J. Endotoxin Res..

[86] Caradonna L., Mastronardi M.L., Magrone T., Cozzolongo R., Cuppone R., Manghisi O.G., Caccavo D., Pellegrino N.M., Amoroso A., Jirillo E. (2002). Biological and clinical significance of endotoxemia in the course of hepatitis C virus infection. Curr. Pharm. Des..

[87] Wu I.W., Lee C.C., Hsu H.J., Sun C.Y., Chen Y.C., Yang K.J., Yang C.W., Chung W.H., Lai H.C., Chang L.C. (2020). Compositional and Functional Adaptations of Intestinal Microbiota and Related Metabolites in CKD Patients Receiving Dietary Protein Restriction. Nutrients.

[88] Di Serio F., Lovero R., D’Agostino D., Nisi LMiragliotta G., Contino R., Man A., Ciccone M.M., Santacroce L. (2016). Evaluation of procalcitonin, vitamin D and C-reactive protein levels in septic patients with positive emocoltures. Our preliminary experience. Acta Med. Mediter..

[89] Zhao J., Ning X., Liu B., Dong R., Bai M., Sun S. (2021). Specific alterations in gut microbiota in patients with chronic kidney disease: An updated systematic review. Ren. Fail..

[90] Merino-Ribas A., Araujo R., Pereira L., Campos J., Barreiros L., Segundo M.A., Silva N., Costa C.F.F.A., Quelhas-Santos J., Trindade F. (2022). Vascular Calcification and the Gut and Blood Microbiome in Chronic Kidney Disease Patients on Peritoneal Dialysis: A Pilot Study. Biomolecules.

[91] Colella M., Topi S., Palmirotta R., D’Agostino D., Charitos I.A., Lovero R., Santacroce L. (2023). An Overview of the Microbiota of the Human Urinary Tract in Health and Disease: Current Issues and Perspectives. Life.

[92] Aragón I.M., Herrera-Imbroda B., Queipo-Ortuño M.I., Castillo E., Del Moral J.S., Gómez-Millán J., Yucel G., Lara M.F. (2018). The Urinary Tract Microbiome in Health and Disease. Eur. Urol. Focus.

[93] Rajpoot M., Sharma A.K., Sharma A., Gupta G.K. (2018). Understanding the microbiome: Emerging biomarkers for exploiting the microbiota for personalized medicine against cancer. Semin. Cancer Biol..

[94] Cho E.J., Leem S., Kim S.A., Yang J., Lee Y.B., Kim S.S., Cheong J.Y., Cho S.W., Kim J.W., Kim S.M. (2019). Circulating Microbiota-Based Metagenomic Signature for Detection of Hepatocellular Carcinoma. Sci. Rep..

[95] Dong Z., Chen B., Pan H., Wang D., Liu M., Yang Y., Zou M., Yang J., Xiao K., Zhao R. (2019). Detection of Microbial 16S rRNA Gene in the Serum of Patients with Gastric Cancer. Front. Oncol..

[96] Woerner J., Huang Y., Hutter S., Gurnari C., Sánchez J.M.H., Wang J., Huang Y., Schnabel D., Aaby M., Xu W. (2022). Circulating microbial content in myeloid malignancy patients is associated with disease subtypes and patient outcomes. Nat. Commun..

[97] Yang D., Wang X., Zhou X., Zhao J., Yang H., Wang S., Morse M.A., Wu J., Yuan Y., Li S. (2021). Blood microbiota diversity determines response of advanced colorectal cancer to chemotherapy combined with adoptive T cell immunotherapy. Oncoimmunology.

[98] Ouaknine Krief J., Helly de Tauriers P., Dumenil C., Neveux N., Dumoulin J., Giraud V., Labrune S., Tisserand J., Julie C., Emile J.F. (2019). Role of antibiotic use, plasma citrulline and blood microbiome in advanced non-small cell lung cancer patients treated with nivolumab. J. Immunother. Cancer.

[99] Plottel C.S., Blaser M.J. (2011). Microbiome and malignancy. Cell Host Microbe.

[100] Kwa M., Plottel C.S., Blaser M.J., Adams S. (2016). The Intestinal Microbiome and Estrogen Receptor-Positive Female Breast Cancer. J. Natl. Cancer Inst..

[101] Parida S., Sharma D. (2019). The Microbiome-Estrogen Connection and Breast Cancer Risk. Cells.

[102] An J., Kil B.J., Kwon H., Kim Y.J. (2022). Analysis of the Impact of the Presence of Phylum Cyanobacteria in the Microbiome of Patients with Breast Cancer on Their Prognosis. J. Clin. Med..

[103] James W.A., Ogunrinde E., Wan Z., Kamen D.L., Oates J., Gilkeson G.S., Jiang W. (2022). A Distinct Plasma Microbiome but Not Gut Microbiome in Patients with Systemic Lupus Erythematosus Compared to Healthy Individuals. J. Rheumatol..

[104] Tomofuji Y., Maeda Y., Oguro-Igashira E., Kishikawa T., Yamamoto K., Sonehara K., Motooka D., Matsumoto Y., Matsuoka H., Yoshimura M. (2021). Metagenome-wide association study revealed disease-specific landscape of the gut microbiome of systemic lupus erythematosus in Japanese. Ann. Rheum. Dis..

[105] Hammad D.B.M., Hider S.L., Liyanapathirana V.C., Tonge D.P. (2020). Molecular Characterization of Circulating Microbiome Signatures in Rheumatoid Arthritis. Front. Cell. Infect. Microbiol..

[106] Kitamura K., Shionoya H., Suzuki S., Fukai R., Uda S., Abe C., Takemori H., Nishimura K., Baba H., Katayama K. (2022). Oral and Intestinal Bacterial Substances Associated with Disease Activities in Patients with Rheumatoid Arthritis: A Cross-Sectional Clinical Study. J. Immunol. Res..

[107] Okumura T., Horiba K., Kamei H., Takeuchi S., Suzuki T., Torii Y., Kawada J.I., Takahashi Y., Ogura Y., Ogi T. (2021). Temporal dynamics of the plasma microbiome in recipients at early post-liver transplantation: A retrospective study. BMC Microbiol..

[108] Din A.U., Hassan A., Zhu Y., Yin T., Gregersen H., Wang G. (2019). Amelioration of TMAO through probiotics and its potential role in atherosclerosis. Appl. Microbiol. Biotechnol..

[109] Becker C., Neurath M.F., Wirtz S. (2015). The Intestinal Microbiota in Inflammatory Bowel Disease. ILAR J..

[110] Goraya M.U., Li R., Mannan A., Gu L., Deng H., Wang G. (2022). Human circulating bacteria and dysbiosis in non-infectious diseases. Front. Cell. Infect. Microbiol..

[111] Jones E., Stentz R., Telatin A., Savva G.M., Booth C., Baker D., Rudder S., Knight S.C., Noble A., Carding S.R. (2021). The Origin of Plasma-Derived Bacterial Extracellular Vesicles in Healthy Individuals and Patients with Inflammatory Bowel Disease: A Pilot Study. Genes.

[112] Caradonna L., Amati L., Lella P., Jirillo E., Caccavo D. (2000). Phagocytosis, killing, lymphocyte-mediated antibacterial activity, serum autoantibodies, and plasma endotoxins in inflammatory bowel disease. Am. J. Gastroenterol..

[113] Jirillo E., Antonaci S. (1985). Spontaneous adherence of Salmonella typhosa to human peripheral blood lymphocytes in typhoid fever. Infection.

[114] Hyun H., Lee M.S., Park I., Ko H.S., Yun S., Jang D.H., Kim S., Kim H., Kang J.H., Lee J.H. (2021). Analysis of Porcine Model of Fecal-Induced Peritonitis Reveals the Tropism of Blood Microbiome. Front. Cell. Infect. Microbiol..

[115] Li Q., Wang C., Tang C., Zhao X., He Q., Li J. (2018). Identification and Characterization of Blood and Neutrophil-Associated Microbiomes in Patients with Severe Acute Pancreatitis Using Next-Generation Sequencing. Front. Cell. Infect. Microbiol..

[116] Desbois A.C., Ciocan D., Saadoun D., Perlemuter G., Cacoub P. (2021). Specific microbiome profile in Takayasu’s arteritis and giant cell arteritis. Sci. Rep..

[117] Xie J., Li Q., Haesebrouck F., Van Hoecke L., Vandenbroucke R.E. (2022). The tremendous biomedical potential of bacterial extracellular vesicles. Trends Biotechnol..

[118] Weber B., Henrich D., Hildebrand F., Marzi I., Leppik L. (2023). The roles of extracellular vesicles in sepsis and systemic inflammatory response syndrome. Shock.

[119] Ñahui Palomino R.A., Vanpouille C., Costantini P.E., Margolis L. (2021). Microbiota-host communications: Bacterial extracellular vesicles as a common language. PLoS Pathog..

[120] Nogués L., Benito-Martin A., Hergueta-Redondo M., Peinado H. (2018). The influence of tumour-derived extracellular vesicles on local and distal metastatic dissemination. Mol. Asp. Med..

[121] Nagakubo T., Tahara Y.O., Miyata M., Nomura N., Toyofuku M. (2021). Mycolic acid-containing bacteria trigger distinct types of membrane vesicles through different routes. iScience.

[122] Schwechheimer C., Kuehn M.J. (2015). Outer-membrane vesicles from Gram-negative bacteria: Biogenesis and functions. Nat. Rev. Microbiol..

[123] Ellis T.N., Leiman S.A., Kuehn M.J. (2010). Naturally produced outer membrane vesicles from Pseudomonas aeruginosa elicit a potent innate immune response via combined sensing of both lipopolysaccharide and protein components. Infect. Immun..

[124] Tulkens J., De Wever O., Hendrix A. (2020). Analyzing bacterial extracellular vesicles in human body fluids by orthogonal biophysical separation and biochemical characterization. Nat. Protoc..

[125] Ahmadi Badi S., Moshiri A., Fateh A., Rahimi Jamnani F., Sarshar M., Vaziri F., Siadat S.D. (2017). Microbiota-Derived Extracellular Vesicles as New Systemic Regulators. Front. Microbiol..

[126] McArthur S. (2023). Regulation of Physiological Barrier Function by the Commensal Microbiota. Life.

[127] Macia L., Nanan R., Hosseini-Beheshti E., Grau G.E. (2019). Host- and Microbiota-Derived Extracellular Vesicles, Immune Function, and Disease Development. Int. J. Mol. Sci..

[128] Mancini F., Rossi O., Necchi F., Micoli F. (2020). OMV Vaccines and the Role of TLR Agonists in Immune Response. Int. J. Mol. Sci..

[129] Taitz J.J., Tan J.K., Potier-Villette C., Ni D., King N.J., Nanan R., Macia L. (2023). Diet, commensal microbiota-derived extracellu-lar vesicles, and host immunity. Eur J Immunol..

[130] Wang X., Lin S., Wang L., Cao Z., Zhang M., Zhang Y., Liu R., Liu J. (2023). Versatility of bacterial outer membrane vesicles in regulating intestinal homeostasis. Sci. Adv..

